# Variation in mitochondrial minichromosome composition between blood-sucking lice of the genus *Haematopinus* that infest horses and pigs

**DOI:** 10.1186/1756-3305-7-144

**Published:** 2014-03-31

**Authors:** Simon D Song, Stephen C Barker, Renfu Shao

**Affiliations:** 1 GeneCology Research Centre, Faculty of Science, Health, Education and Engineering, University of the Sunshine Coast, Maroochydore, Queensland 4556, Australia; 2 Parasitology Section, School of Chemistry and Molecular Biosciences, The University of Queensland, Queensland 4072, Australia

**Keywords:** Mitochondrial genome, Genome fragmentation, Minichromosome, Chromosome evolution, Sucking lice, Horse louse

## Abstract

**Background:**

The genus *Haematopinus* contains 21 species of blood-sucking lice, parasitizing both even-toed ungulates (pigs, cattle, buffalo, antelopes, camels and deer) and odd-toed ungulates (horses, donkeys and zebras). The mitochondrial genomes of the domestic pig louse, *Haematopinus suis*, and the wild pig louse, *Haematopinus apri*, have been sequenced recently; both lice have fragmented mitochondrial genomes with 37 genes on nine minichromosomes. To understand whether the composition of mitochondrial minichromosomes and the gene content and gene arrangement of each minichromosome are stable within the genus, we sequenced the mitochondrial genome of the horse louse, *Haematopinus asini*.

**Methods:**

We used a PCR-based strategy to amplify four mitochondrial minichromosomes in near full-length, and then amplify the entire coding regions of all of the nine mitochondrial minichromosomes of the horse louse. These amplicons were sequenced with an Illumina Hiseq platform.

**Results:**

We identified all of the 37 mitochondrial genes typical of bilateral animals in the horse louse, *Haematopinus asini*; these genes are on nine circular minichromosomes. Each minichromosome is 3.5–5.0 kb in size and consists of a coding region and a non-coding region except *R-nad4L-rrnS-C* minichromosome, which contains two coding regions and two non-coding regions. Six of the nine minichromosomes of the horse louse have their counterparts in the pig lice with the same gene content and gene arrangement. However, the gene content and arrangement of the other three minichromosomes of the horse louse, including *R-nad4L-rrnS-C*, are different from that of the other three minichromosomes of the pig lice.

**Conclusions:**

Comparison between the horse louse and the pig lice revealed variation in the composition of mitochondrial minichromosomes within the genus *Haematopinus*, which can be accounted for by gene translocation events between minichromosomes. The current study indicates that inter-minichromosome recombination plays a major role in generating the variation in the composition of mitochondrial minichromosomes and provides novel insights into the evolution of fragmented mitochondrial genomes in the blood-sucking lice.

## Background

*Haematopinus* (Leach, 1815) is the only genus in the family Haematopinidae of the suborder Anoplura, known as the blood-sucking lice [1-3]. There are 21 species in the genus *Haematopinus*, of which 19 species parasitize even-toed ungulates (order Artiodactyla) such as pigs, cattle, buffalo, antelopes, camels and deer, whereas the other two species parasitize odd-toed ungulates (order Perissodactyla) such as horses, donkeys and zebras [1,4]. *Haematopinus* species are vectors of several severe infectious diseases in rural tropical areas such as African swine fever [5-7], swinepox [8], hog cholera and eperythrozoonosis [9,10], and anaplasmosis [11].

The typical mitochondrial (mt) genome organization of insects and other bilateral animals consists of a single circular chromosome, 13–20 kb in size, with 37 genes and a control region [12,13]. Fragmented mt genomes, however, have been found in five species of blood-sucking lice: human body louse, *Pediculus humanus*, human head louse, *Pe. capitis*, human pubic louse, *Pthirus pubis,* domestic pig louse, *Haematopinus suis* and the wild pig louse, *H. apri*[14-16]. The extent of mt genome fragmentation, the number of minichromosomes and the distribution of genes on the minichromosomes vary remarkably between genera of the blood-sucking lice, but are the same for the species within the same genus [14-16]. For instance, both the human body louse, *Pe. humanus*, and the human head louse, *Pe. capitis*, have 20 mt minichromosomes and an identical pattern for the distribution of mt genes on these minichromosomes [14,16]. The domestic pig louse, *H. suis*, and the wild pig louse, *H. apri*, have nine mt minichromosomes and an identical pattern for the distribution of mt genes too [15]. The human head louse and the human body louse, however, are very closely related and had their most recent common ancestor (MRCA) ~107,000 years ago [17,18]; so are the domestic pig louse and the wild pig louse, which had their MRCA ~9,000 years ago [19].

To understand whether the composition of mt minichromosomes, and the gene content and gene arrangement in each minichromosome are indeed conserved among species of the same genus, we sequenced the mt genome of the horse louse, *Haematopinus asini*, and compared it with those of the pig lice, *H. suis* and *H. apri. H. asini* parasitizes horses (*Equus caballus*) and two other odd-toed ungulates: donkeys (*E. asinus*) and plains zebras (*E. burchelli*) [20]. We found that the horse louse differs from the pig lice in the distribution of mt genes on three of the nine minichromosomes and the intra-genus variation can be explained by gene translocation between minichromosomes.

## Methods

### Sample collection, DNA extraction, PCR amplification and DNA sequencing

**Figure 1:**
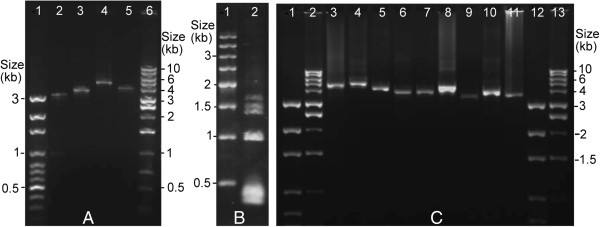
**PCR amplicons from the mitochondrial genome of the horse louse, *Haematopinus asini*. (A)** Amplicons generated with the horse-louse-specific primers, 12sB2448F–12sB2448R (lane 2), 16sB2448F–16sB2448R (lane 3), cox1B2448F–cox1B2448R (lane 4) and cox2B2448F–cox2B2448R (lane 5) from four mitochondrial minichromosomes. Lane 1 and lane 6: 100-bp Ladder and 1-kb Ladder (BioSciences). **(B)** Amplicons generated with the primer pair B2448F-B2448R from the coding regions of all of the mitochondrial minichromosomes of the horse louse (lane 2). Lane 1: 500-bp DNA Ladder (Tiangen). **(C)** PCR verification of the mt minichromosomes of the horse louse. Lane 1 and 12: 100-bp ladder. Lane 2 and 13: 1-kb ladder. Lane 3–11: PCR amplicons from the nine minichromosomes of the horse louse: *K-****nad4****-atp8-atp6-N*, ***nad2****-I-cox1-L*_*2*_, *D-Y-****cox2****-S*_*1*_*-S*_*2*_*-P-cox3-A*, *E-****cob****-V*, *Q-nad1-T-G-****nad3****-W*, *H-****nad5****-F-nad6*, ***M***, ***L***_***1***_*-rrnL* and *R-nad4L-****rrnS****-C*. Genes from which PCR primers were designed are in bold.

Specimens of *H. asini* were collected from horses at Kemps Creek, Sydney, Australia in 2008 (sample # B2448). The study does not involve the ethical treatment of horse, other than collecting lice from their body surface. Genomic DNA was extracted from individual louse specimens with DNeasy Tissue kit (QIAGEN). Four pairs of primers, 12SA–12SB, 16SF–Lx16SR, mtd6–mtd11 and mtd16–mtd18, were used to amplify fragments of *rrnS* (350 bp), *rrnL* (300 bp), *cox1* (600 bp) and *cox2* (230 bp) (see Additional file 1). These fragments were sequenced with AB3730xl sequencers. Four pairs of outbound primers (forward and reverse), 12sB2448F–12sB2448R, 16sB2448F–16sB2448R, cox1B2448F–cox1B2448R and cox2B2448F–cox2B2448R (see Additional file 1), were designed from the sequences of the *rrnS*, *rrnL*, *cox1* and *cox2* fragments, respectively. PCRs with these specific primers amplified four mt minichromosomes in near full-length that contained each of the four genes, 3.5 kb, 3.8 kb, 5.0 kb and 4.1 kb in size respectively (Figure 1A). These amplicons were sequenced partially with AB3730xl sequencers. Sequences from the non-coding regions of these four minichromosomes were aligned with Clustal X [21]. A forward primer B2448F and a reverse primer B2448R were designed from conserved motifs in the non-coding regions that flank the coding regions of the four minichromosomes above (see Additional file 1). A mixture of PCR amplicons ranging from 350 bp to 2,900 bp was obtained with the primer pair B2448F–B2448R; these amplicons were expected from the coding regions of all mt minichromosomes of the horse louse (Figure 1B). Amplicons generated with B2448F–B2448R and those with primer pairs 12sB2448F–12sB2448R, 16sB2448F–16sB2448R, cox1B2448F–cox1B2448R and cox2B2448F–cox2B2448R were sequenced with Illumina Hiseq 2000 platform at the BGI, Hong Kong.

Takara Ex *Taq* was used in the initial short PCRs with the following cycling conditions: 94°C for 1 min; 35 cycles of 98°C for 10 sec, 45°C for 30 sec, 72°C for 1 min; and a final extension of 72°C for 2 min. TaKaRa LA *Taq* was used in the long PCRs with the cycling conditions: 94°C for 1 min, 35 cycles of 98°C for 10 sec, 55–65°C (depending on primers) for 40 sec, 68°C for 4 min; and 72°C for 8 min. Negative controls were executed with each PCR experiment. PCR amplicons were checked by agarose gel (1%) electrophoresis; the sizes of PCR amplicons were estimated by comparison with molecular markers. Wizard SV Gel and PCR Clean-up System (Promega) were used to purify PCR amplicons for sequencing.

### Assembly of Illumina sequence-reads, gene identification and verification of mitochondrial minichromosomes

Illumina sequence-reads were assembled *de novo* with Geneious (Version 6.1.6, Biomatters); the assembly parameters were 98% and 50 bp for minimum overlap identity and minimum overlap, respectively. tRNA genes were identified using program tRNAscan-SE [22] and ARWEN [23]. Protein-coding genes and rRNA genes were identified with BLAST searches of GenBank [24,25]. Identical sequences shared between genes were searched with program Wordmatch [26]. The length of identical sequences shared by chance between genes was assessed by analyzing randomly-extracted, unrelated DNA sequences from GenBank of the same sizes as our experimental sequences. Take tRNA genes, which are ~ 70 nt in length, as an example. We extract, randomly, a set of unrelated DNA sequences (n > 50) from GenBank; each of these sequences is 70 nt. We then run these sequences in Wordmatch and identify the size of the longest identical sequences shared between any pairs of the extracted sequences. We work out the average (~7 nt in the case of tRNA genes) and use it as an indication of chance expectation of the length of identical sequences for tRNA genes.

The size and circular organization of each mt minichromosome identified by sequence-read assembly were verified by PCR. A pair of outbound primers (forward and reverse) was designed from the coding region of each minichromosome (see Additional file 2). PCRs with these primers amplified each minichromosome in full-length or near full-length if the minichromosomes had a circular organization. The amplicons generated with primer pairs nad4f–nad4r, cobf–cobr, nad3f–nad3r, nad5f–nad5r and metf–metr were also sequenced with Illumina Hiseq 2000 platform. PCR set-up, cycling conditions, agarose gel electrophoresis and size measurement were the same as the long PCRs described above.

## Results and Discussion

### Mitochondrial genome of the horse louse, *Haematopinus asini*

**Figure 2:**
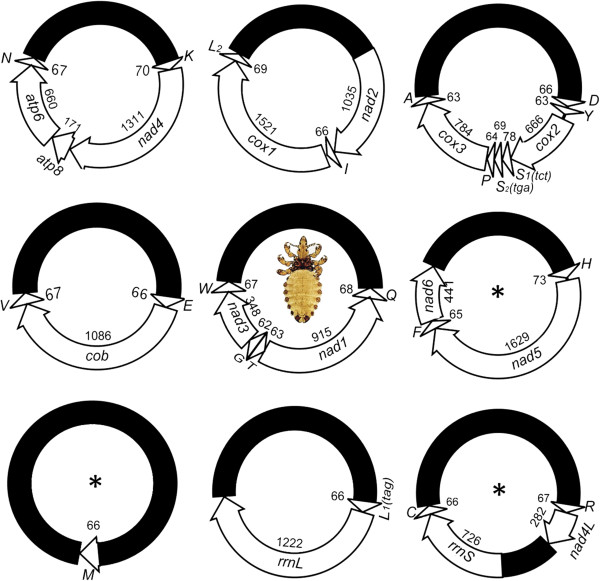
**Mitochondrial genome of the horse louse, *Haematopinus asini*.** The name, transcription orientation and length (bp) of each gene are indicated. Non-coding regions are in black. Abbreviations of gene names are: *cox1*–*3* for cytochrome c oxidase subunits 1–3; *cob* for cytochrome b; *nad1*–*5* and *nad4L* for NADH dehydrogenase subunits 1–5 and 4 L; and *rrnS* and *rrnL* for small and large ribosome RNA subunits. tRNA genes are labeled with the single-letter abbreviations of their corresponding amino acids. Numbers indicate the length of each corresponding gene. Minichromosomes shown with asterisk symbols (*) have different gene content and gene arrangement compared with the pig lice, *Haematopinus suis* and *Haematopinus apri*[15].

We obtained 897,097 Illumina sequence-reads (pair end, 180-bp inserts) from the amplicons of the mt genome of the horse louse, *H. asini* (Table 1). The sequence-reads are all 90 bp each in length. We assembled these sequence-reads into contigs and identified all of the 37 mt genes typical of bilateral animals in *H. asini,* distributed on nine circular minichromosomes (Figure 2; Figure 1C). The mt minichromosomes of the horse louse are 3.5–5.0 kb in size (Figure 1C) and are the largest among those of the sucking lice known, due to their expanded non-coding regions (see below). Each minichromosome of the horse louse consists of a coding region and a non-coding region except the *R-nad4L-rrnS-C* minichromosome, which has two coding regions and two non-coding regions (Figure 2). There are 1–8 genes in each coding region, varying in size from 66 bp for *trnM* minichromosome to 2,699 bp for *nad2-trnI-cox1-trnL*_*2*_ minichromosome (Table 1). With the exception of *trnT, nad1* and *trnQ*, all of the mt genes have the same orientation of transcription relative to the non-coding region (Figure 2). The nucleotide sequences of the mt minichromosomes of *H. asini* were deposited in GenBank under accession numbers KF939318, KF939322, KF939324, KF939326 and KJ434034-KJ434038 (Table 1).

**Table 1:** **Mitochondrial minichromosomes of the horse louse, *Haematopinus asini*, identified by Illumina sequencing**

**Minichromosome**	**GenBank accession number**	**Size of coding region (bp)**	**Number of Illumina sequence-reads**	**Mean coverage**
*D-Y-cox2-S*_*1*_*-S*_*2*_*-P-cox3-A*	KF939318	1871	150403	3420
*E-cob-V*	KJ434038	1218	281565	6435
*H-nad5-F-nad6*	KJ434037	2229	84549	1579
*K-nad4-atp8-atp6-N*	KJ434035	2282	1009104	18660
*L*_*1*_*-rrnL*	KF939322	1289	120832	7590
*M*	KJ434036	66	409434	10790
*nad2-I-cox1-L*_*2*_	KF939324	2699	133014	2990
*Q-nad1-T-G-nad3-W*	KJ434034	1505	487487	8846
*R-nad4L-rrnS-C*	KF939326	1142	16,059	385
Total		14301	2692447	60695

**Figure 3:**
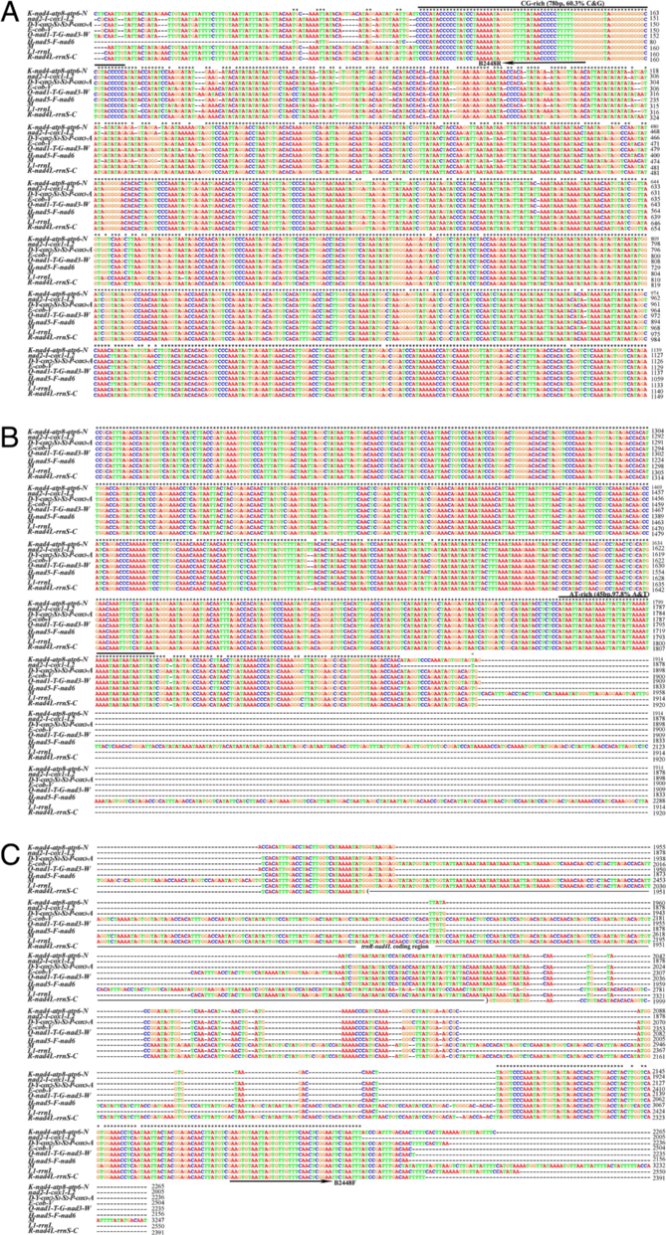
**Alignment of the full-length non-coding regions of nine mitochondrial minichromosomes of the horse louse, *Haematopinus asini*.** B2448F and B2448R are the primers used to amplify the entire coding regions of all mitochondrial minichromosomes of the horse louse.

We sequenced the non-coding regions of all of the nine mt minichromosomes of the horse louse in full length, which range from 2,005 bp to 3,264 bp (Figure 3). The horse louse is the first species of sucking lice for which the full-length non-coding regions of all mt minichromosomes were sequenced. The horse louse has the longest non-coding regions among the sucking lice known; previously the longest non-coding region was 2,370 bp, noted in the pig lice [15]. As in the human lice and the pig lice, there is an AT-rich motif (45 bp, 100% A and T) in the non-coding region upstream the 5′-end of coding region and a GC-rich motif (78 bp, 60% C and G) downstream the 3′-end of the coding region (Figure 3). The size variation among the nine non-coding regions of the horse louse is due to size variation in the section upstream the coding region from the AT-rich motif to the primer B2448F (Figure 3). Excluding this section, the non-coding regions of the minichromosomes have ~ 96% pairwise identity to each other.

### Shared identical sequences and recombination between mt genes in the horse louse

Ten pairs of mitochondrial genes share stretches of identical sequences longer than expected by chance in the blood-sucking lice of humans and pigs, providing unequivocal evidence for DNA recombination between mt genes and between minichromosomes in these lice [14,16]. We found that nine pairs of mt genes in the horse louse, *H. asini*, also share stretches of identical sequences longer than expected by chance (Table 2). *trnL*_*1*_ and *trnL*_*2*_ are the only pair of genes that share longer than expected identical sequences in all of the three species of human lice and the two species of pig lice, indicating recombination between these two genes has been maintained since their MRCA, ~ 65 million years ago (Mya) [27]. This is also the case for the horse louse although the 15-bp identical sequence shared between these two genes is the shortest among the sucking lice known. Any two non-homologous tRNA genes might be expected to share identical sequences, ~ 7 bp in size, by chance; the identical sequence shared between *trnL*_*1*_ and *trnL*_*2*_ in the horse louse is twice as long as expected by chance (Table 2; see Additional file 3). As in the two pig lice, *trnT* and *trnP* in the horse louse share 27-bp identical sequence, which is four times as long as expected by chance (Table 2; see Additional file 3). In the three human lice and other animals, *trnT* and *trnP* share identical sequences, 6–9 bp long, which are expected by chance. Recombination between *trnT* and *trnP*, thus, is likely a derived feature for the genus *Haematopinus*. Seven other pairs of mt genes also share identical sequences 1.5–3 times longer than expected by chance in the horse louse but not in the pig lice nor in the human lice (Table 2). Recombination between these seven pairs of genes, therefore, is likely a derived feature for the horse louse only (Table 2).

**Table 2:** **The longest stretches of identical sequence shared between mitochondrial genes in the horse louse, *Haematopinus asini***

		**The longest stretches of identical sequence shared (bp)**									
**Pairs of genes**		**Horse louse**	**Pig lice**		**Human lice**			**Animals with typical mt genome organization**			
		***Haas***	***Has***	***Haap***	***Peh***	***Pec***	***Ptp***	***Bm***	***Cb***	***Hm***	***Dy***
*cob*	*cox1*	**21**	11	11	10	10	10	12	13	14	13
*nad2*	*nad4*	**20**	9	12	14	14	N/A	12	16	13	12
*nad4*	*cob*	**20**	10	10	11	11	N/A	10	12	12	12
*nad2*	*nad5*	**25**	10	10	14	14	10	12	16	12	15
*nad2*	*nad3*	**19**	12	12	10	10	10	10	12	11	11
*rrnL*	*rrnS*	**10, 33**	10	9	11	11	10	**33**	10	15	16
*rrnL*	*trnN*	**15**	9	9	9	9	N/A	10	8	9	8
*trnL*_*1*_	*trnL*_*2*_	**15**	**16,10,9**	**16,10,9**	**33,32**	**33,32**	**35,32**	7	6	7	10
*trnT*	*trnP*	**27**	**26**	**26**	7	7	N/A	6	8	8	9

Note: Abbreviations of species names are: *Haas*, *Haematopinus asini* (horse louse); *Has*, *Haematopinus suis* (domestic pig louse); *Haap*, *Haematopinus apri* (wild pig louse); *Peh, Pediculus humanus* (human body louse); *Pec, Pediculus capitis* (human head louse); *Ptp, Pthirus pubis* (human pubic louse); *Bm, Bothriometopus macrocnemis* (screamer louse); *Cb, Campanulotes bidentatus* (pigeon louse); *Hm, Heterodoxus macropus* (wallaby louse); *Dy, Drosophila yakuba* (fruitfly); *N/A*, Not available. *mt*, Mitochondrial; Stretches of shared identical sequences longer than expected by chance are in bold.

### Variation in mt minichromosome composition between the horse louse and the pig lice

Six of the nine minichromosomes of the horse louse, *H. asini*, have the same gene content and gene arrangement as their counterparts of the pig lice, *H. suis* and *H. apri* (Figure 2) [15]. These minichromosomes are apparently ancestral to *Haematopinus* species and thus have been retained in both the horse louse and the pig lice. The other three minichromosomes of the horse louse, however, are not present in the pig lice (Figure 2). In the pig lice, one of the minichromosomes has three genes, *trnH-nad5-trnF*[15]. the horse louse, however, the minichromosome that has these three genes also has *nad6* gene downstream *trnF* with a gap of 3 bp in between (Figure 2). Similarly, another minichromosome of the pig lice has two genes, *rrnS-trnC*. In the horse louse, however, the minichromosome that has these two genes has *trnR-nad4L* upstream *rrnS* with a 417-bp non-coding region in between (Figure 2). Furthermore, in the pig lice*, trnM* gene is on a minichromosome with *nad6* and *trnR-nad4L*[15]. In the horse louse, however, *trnM* is alone on its own minichromosome (Figure 2).

### How was the intra-genus variation in mt minichromosome composition generated?

Our comparison of the mt genomes of the horse louse and the pig lice revealed variation in the composition of mt minichromosomes within the genus *Haematopinus*. Several previous studies also compared mt genomes between species of sucking lice in the same genus. The human head louse and the human body louse in the genus *Pediculus* have identical mt minichromosome composition [17,18], so are the domestic pig louse and the wild pig louse in the genus *Haematopinus*[19]. The current study compared *Haematopinus* species that infest mammals distinct from one another. *Haematopinus asini* parasitizes exclusively horses, donkeys and zebras, which are odd-toed ungulates (order Perissodactyla). *Haematopinus suis* and *H. apri*, however, parasitize exclusively domestic pigs and wild pigs, which are even-toed ungulates (order Artiodactyla). These two lineages of ungulate mammals had their MRCA 63–83 Mya [28-30].

**Figure 4:**
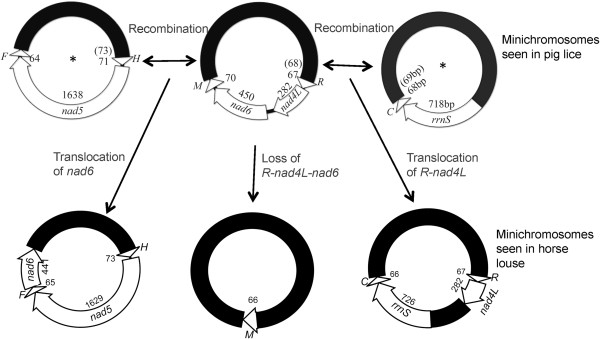
**An inter-minichromosome recombination model that accounts for the variation in the composition of mitochondrial minichromosomes between the horse louse, *Haematopinus asini*, and the pig lice, *H. suis *and *H. apri*.** Minichromosomes shown with asterisk symbols (*) were seen in a *Polyplax* rat louse [31].

A very recent study by Dong et al. showed that two species of rat lice in the genus *Polyplax* also differ in the composition of mt minichromosomes [31]. Together, these studies indicate that intra-genus variation in mt minichromosome composition is likely common in blood-sucking lice. Furthermore, these studies provided opportunities to look into how fragmented mt genomes evolved in the blood-sucking lice. The typical single-chromosome mt genomes are highly conserved in genome organization, gene content and gene arrangement in the vast majority of insects [12,32]. The fragmented mt genomes of the sucking lice known to date, however, showed very limited conservation in terms of the number of minichromosomes, and the gene content and gene arrangement in each minichromosome. The current study indicates that inter-minichromosome recombination plays a major role in the fast evolution of fragmented mt genomes in the blood-sucking lice [33]. The variation between the horse louse and the pig lice in mt minichromosome composition can be accounted for parsimoniously by two events of inter-minichromosome recombination. Firstly, a recombination event translocated *R-nad4L* from a minichromosome that contained *R-nad4L-nad6-M* (a minichromosome seen in the pig lice, [15]) to a minichromosome that contained *rrnS-trnC* and generated a minichromosome with two coding regions and two non-coding regions in the horse louse: *R-nad4L* in one coding region whereas *rrnS-trnC* in another coding region (Figure 4). The minichromosome that contained *rrnS-trnC* was seen in the *Haematopinus* pig lice [15] and a *Polyplax* rat louse [31], and thus can be inferred to be ancestral to *Haematopinus* species. Secondly, another recombination event translocated *nad6* from the *R-nad4L-nad6-M* minichromosome to a minichromosome that contained *trnH-nad5-trnF*, and generated a minichromosome with both *nad6* and *trnH-nad5-trnF* in the horse louse (Figure 4). The minichromosome that contained *trnH-nad5-trnF* was seen in the *Haematopinus* pig lice [15] and a *Polyplax* rat louse [31], and can be inferred to be ancestral to *Haematopinus* species. Finally, the loss of *R-nad4L* and *nad6* from *R-nad4L-nad6-M* minichromosome led to a minichromosome with only *trnM*, seen in the horse louse (Figure 4).

## Conclusions

We sequenced the mt genome of the horse louse, *H. asini*. We found all of the 37 mt genes typical of insects and other bilateral animals; these genes are on nine circular minichromosomes. Each minichromosome is 3.5–5.0 kb in size and contains 1–8 genes. Three of the nine minichromosomes of the horse louse differ from those of the pig lice in gene content and gene arrangement, revealing variation in the composition of mt minichromosomes among species of the genus *Haematopinus*. We propose that inter-minichromosome recombination can cause gene translocations and likely plays a major role in generating the variation in the composition of mt minichromosomes observed in the *Haematopinus* and other blood-sucking lice.

## Abbreviations

MRCA: Most recent common ancestor; Mya: Million years ago; mt: Mitochondrial.

## Competing interests

The authors declare that they have no competing interests.

## Authors’ contributions

SS, SCB and RS conceived and designed the research. SS conducted the research. SS and RS analyzed and interpreted the data. SCB and RS contributed reagents and materials. SS and RS wrote the manuscript. All authors read and approved the submitted version of the manuscript.

## Supplementary Material

Additional file 1 **PCR primers used to amplify and sequence the mitochondrial genome of the house louse, *Haematopinus asini*.** Click here for file

Additional file 2 **PCR primers used to verify each mitochondrial minichromosome of the house louse, *Haematopinus asini*.** Click here for file

Additional file 3 **The inferred secondary structure of the mitochondrial tRNAs of the horse louse, *Haematopinus asini*.** Shared identical sequences between tRNA genes are in bold (see also Table 2). Click here for file

## References

[B1] Durden LA Musser GG The sucking lice (Insecta, Anoplura) of the world: a taxonomic checklist with records of mammalian hosts and geographical distributions Bull Am Mus Nat Hist 1994 218 1 90

[B2] Meleney WP Kim KC A comparative study of cattle-infesting *Haematopinus*, with redescription of *H. quadripertusus* Fahrenholz, 1916 (Anoplura: Haematopinidae) J Parasitol 1974 60 507 522 10.2307/3278373 4857799

[B3] Barker SC Phylogeny and classification, origins, and evolution of host associations of lice Int J Parasitol 1994 8 1285 1291 7729981 10.1016/0020-7519(94)90195-3

[B4] Scofield A Campos KF Silva AMM Oliveira CHS Barbosa JD Góes-Cavalcante G Infestation by *Haematopinus quadripertusus* on cattle in São Domingos do Capim, state of Pará, Brazil Rev Bras Parasitol Vet Revista 2012 21 315 318 10.1590/S1984-29612012000300027 23070449

[B5] Seifert HSH Tropical Animal Health 1996 Dordrecht, the Netherlands: Kluwer Academic Publishers 572

[B6] Wall R Shearer D Wall R, Shearer D Lice (Phthiraptera) Veterinary Ectoparasites: Biology, Pathology and Control 2008 2 Oxford, UK: Blackwell Science Ltd 162 178

[B7] Penrith ML Vosloo W Review of African swine fever: transmission, spread and control Oorsigartikel 2009 80 58 62 10.4102/jsava.v80i2.172 19831264

[B8] Thibault S Drolet R Alain R Dea S A sporadic skin disorder in nursing piglets Swine Health Prod 1998 6 276 278

[B9] Agbede RIS A survey of ectoparasites and parasitic conditions of animals in Zaria Nigerian J Anim Prod Res 1981 1 179 180

[B10] Portiansky EL Auiroga MA Machuca MA Perfumo CJ *Mycoplasma suis* in naturally infected pigs: an ultrastructural and morphometric study Pesquisa Vet Brasil 2004 24 1 5 10.1590/S0100-736X2004000100002

[B11] Da-Silva A Lopes L Diaz J Tonin A Stefani L Araújo D Lice outbreak in buffaloes: Evidence of *Anaplasma marginale* transmission by sucking lice *Haematopinus tuberculatus* J Parasitol 2013 99 546 547 10.1645/GE-3260.1 23050728

[B12] Boore J Animal mitochondrial genomes Nucleic Acids Res 1999 27 1767 1870 10.1093/nar/27.8.1767 10101183 PMC148383

[B13] Lavrow D Key transitions in animal evolution: a mitochondrial DNA perspective Integr Comp Biol 2007 47 734 743 10.1093/icb/icm045 21669754

[B14] Shao R Kirkness EF Barker SC The single mitochondrial chromosome typical of animals has evolved into 18 minichromosomes in the human body louse, *Pediculus humanus* Genome Res 2009 19 904 912 10.1101/gr.083188.108 19336451 PMC2675979

[B15] Jiang H Barker SC Shao R Substantial variation in the extent of mitochondrial genome fragmentation among blood-sucking lice of mammals Genome Biol Evol 2013 5 1298 1308 10.1093/gbe/evt094 23781098 PMC3730346

[B16] Shao R Zhu XQ Barker SC Herd K Evolution of extensively fragmented mitochondrial genomes in the lice of humans Genome Biol Evol 2012 4 1088 1101 10.1093/gbe/evs088 23042553 PMC3514963

[B17] Kittler R Kayser M Stoneking M Molecular evolution of *Pediculus humanus* and the origin of clothing Curr Biol 2004 13 1414-1417 12932325 10.1016/s0960-9822(03)00507-4

[B18] Kittler R Kayser M Stoneking M Molecular evolution of Pediculus humanus and the origin of clothing (Erratum) Curr Biol 2004 14 2309 10.1016/j.cub.2004.12.024 12932325

[B19] Giuffra D Kijas JMH Amarger V Carlborg O Jeon JT Andersson L The origin of the domestic pig: independent domestication and subsequent introgression Genetics 2000 154 1785 1791 10747069 10.1093/genetics/154.4.1785 PMC1461048

[B20] Durden LA Musser GG The mammalian hosts of the sucking lice (Anoplura) of the world: a host-parasite list Bull Soc Vector Ecol 1994 19 130 168

[B21] Larkin MA Blackshields G Brown NP Chenna R McGettigan PA McWilliam H Valentin F Wallace IM Wilm A Lopez R Thompson JD Gibson TJ Higgins DG Clustal W and Clustal X version 2.0 Bioinformatics 2007 23 2947 2948 10.1093/bioinformatics/btm404 17846036

[B22] Lowe TM Eddy SR tRNAscan-SE: A program for improved detection of transfer RNA genes in genomic sequence Nucleic Acids Res 1997 25 955 964 10.1093/nar/25.5.0955 9023104 PMC146525

[B23] Laslett D Canbäck B ARWEN, a program to detect tRNA genes in metazoan mitochondrial nucleotide sequences Bioinformatics 2008 24 172 175 10.1093/bioinformatics/btm573 18033792

[B24] McGinnis S Madden TL BLAST: At the core of a powerful and diverse set of sequence analysis tools Nucleic Acids Res 2004 32 W20 W25 10.1093/nar/gkh435 15215342 PMC441573

[B25] Zheng Z Schaffer AA Miller W Madden TL Lipman DJ Koonin EV Altschul SF Protein sequence similarity searches using patterns as seeds Nucleic Acids Res 1998 26 3986 3990 10.1093/nar/26.17.3986 9705509 PMC147803

[B26] Rice P Longden I Bleasby A EMBOSS: the European molecular open software suite Trends in Genet 2000 16 276 277 10.1016/S0168-9525(00)02024-2 10827456

[B27] Light JE Smith VS Allen JM Durden LA Reed DL Evolutionary history of mammalian sucking lice (Phthiraptera: Anoplura) BMC Evol Biol 2010 10 292 10.1186/1471-2148-10-292 20860811 PMC2949877

[B28] Cornelis G Heidmann O Degrelle SA Vernochet C Lavialle C Letzelter C Bernard-Stoecklin S Hassanin A Mulot B Guillomot M Hue I Heidmann T Dupressoir A Captured retroviral envelope syncytin gene associated with the unique placental structure of higher ruminants Proc Natl Acad Sci U S A 2013 110 E828 E837 10.1073/pnas.1215787110 23401540 PMC3587263

[B29] Murphy WJ Pringle TH Crider TA Springer MS Miller W Using genomic data to unravel the root of the placental mammal phylogeny Genome Res 2007 17 413 421 10.1101/gr.5918807 17322288 PMC1832088

[B30] Novacek MJ Prothero DR, Schock RM The radiation of placental mammals Major Features of Vertebrate Evolution 1994 Knoxville: Paleontological Society Short Courses in Paleontology and University of Tennessee Press 220 237

[B31] Dong WG Song S Jin DC Guo XG Shao R Fragmented mitochondrial genomes of the rat lice, Polyplax asiatica and Polyplax spinulosa: intra-genus variation in fragmentation pattern and a possible link between the extent of fragmentation and the length of life cycle BMC Genomics 2014 15 44 10.1186/1471-2164-15-44 24438034 PMC3901344

[B32] Wolstenholme DR Genetic novelties in mitochondrial genomes of multicellular animals Curr Opin Genet Dev 1992 2 919 925 10.1016/s0959-437x(05)80116-9 1282405

[B33] Shao R Barker SC Chimeric mitochondrial minichromosomes of the human body louse, *Pediculus humanus*: Evidence for homologous and non-homologous recombination Gene 2011 473 36 43 10.1016/j.gene.2010.11.002 21092752

