# Supplementary material for: Variation in mitochondrial minichromosome composition between blood-sucking lice of the genus Haematopinus that infest horses and pigs
Source: Parasit Vectors. 2014 Mar 31;7:144. doi: 10.1186/1756-3305-7-144 (PMC4022054; doi:10.1186/1756-3305-7-144)
Supplement: Additional file 1 — PCR primers used to amplify and sequence the mitochondrial genome of the house louse, Haematopinus asini . [file 1756-3305-7-144-S1.pdf]

Additional file 1 PCR primers used to amplify and sequence the mitochondrial genome of the house louse, *Haematopinus asini*

Primer	Sequence (5' to 3')	Target gene or region
12SA	TACTATGTTACGACTTAT	*rrnS*
12SB	AAACTAGGATTAGATACCC	*rrnS*
16SF	TTAATTCAACATCGAGGTCGCAA	*rrnL*
Lx16SR	GACTGTGCTAAGGTAGCATAAT	*rrnL*
mtd6	GGAGGATTTGGAAATTGATTAGTTCC	*cox1*
mtd11	ACTGTAAATATATGATGAGCTCA	*cox1*
mtd16	ATTGGACATCAATGATATTGA	*cox2*
mtd18	CCACAAATTTCTGAACATTGACCA	*cox2*
12sB2448F	GTCAAGTCAAGGTGCAGACGATAACAAGGC	*rrnS*
12sB2448R	CACATTACAACCTTCCGACAGCGGTGTACA	*rrnS*
16sB2448F	CTATTTACTTGGGAAGAGGGCTTAAAGGA	*rrnL*
16sB2448R	TCTATAGGGTCTTCTCGTCCTGCTAGAC	*rrnL*
cox1B2448F	AGAGGAGGATACACTGTTCAACCAGTCCC	*cox1*
cox1B2448R	GCCAACCAGGCTGTTCTGTTGACTTAGC	*cox1*
cox2B2448F	GCTGTCATACCTCTCATCTGACCAATCAGTG	*cox2*
cox2B2448R	GTTCGGCTGTTAGTTACTTCTTCGGATGTG	*cox2*
B2448F	TCGAAGTGTAATTAGTGTTGTTTCAACTCGGAA	NCR
B2448R	CCCTATTTTTAGGATAGGGGGTATGGGAA	NCR

Note: Abbreviations of target gene or region names are: *rrnS* and *rrnL* for small and large ribosome RNA subunits; *cox1-2* for cytochrome c oxidase subunits 1-2; and NCR for no-coding region.
