# Supplementary material for: Variation in mitochondrial minichromosome composition between blood-sucking lice of the genus Haematopinus that infest horses and pigs
Source: Parasit Vectors. 2014 Mar 31;7:144. doi: 10.1186/1756-3305-7-144 (PMC4022054; doi:10.1186/1756-3305-7-144)
Supplement: Additional file 2 — PCR primers used to verify each mitochondrial minichromosome of the house louse, Haematopinus asini . [file 1756-3305-7-144-S2.pdf]

Additional file 2 PCR primers used to verify each mitochondrial minichromosome of the house louse, *Haematopinus asini*

Primer	Sequence (5' to 3')	Target gene
nad4f	GATAGACAATACTTATTGGGGCACGTTTCGCA	*nad4*
nad4r	GCAGACTCCGCTATTATTTAGTCTCACAACC	*nad4*
nad2f	TATGGAAAGTTAGGTAGTCTAGGGT	*nad2*
nad2r	GAAAGAAGTTAACTCCATACCGAAC	*nad2*
cox2f	TGAAGAGAGATGCTATTCCGGGTCG	*cox2*
cox2r	AATCACATCCGAAGAAGTAACTAACAGCCGAA	*cox2*
cobf	TAACGGCTTGAATTTGAGGTGGGT	*cob*
cobr	ATGCGCCTCAGTACGGTAAAGC	*cob*
nad3f	ATATGAGGCTCACTAGATTGGGACAAA	*nad3*
nad3r	TGCCCACGAAAAGGATCAATCTGTAGA	*nad3*
nad5f	GGAGGACTAGTATAGAAACTATGTCTTTGGGAGG	*nad5*
nad5r	GGAGACTACTAGCAATGGGAATTTGGAGAC	*nad5*
metf	CATACTCTAACGAAATGTAACACATTTTGCTTCAA	*trnM*
metr	AAGGCTTAAGTTATAGCCTATTTGCTCGA	*trnM*
trnlf	GGATTTAGGATTCAAATATGATGTTTATCTTCAC	*trnL1*
trnlr	ATTCTGCCAACCTAACTTATATGGAAAC	*trnL1*
12sf	AGAGTATGGCGGTCAATATCCTGATCAGAGACTC	*rrnS*
12sr	TGGAGTAACCCAATACTTCCTTGGTTTAGGGGCA	*rrnS*

Note: Abbreviations of gene names are: *nad2*-5 for NADH dehydrogenase subunits 2-5; *cox2* for cytochrome c oxidase subunits 2; *cob* for cytochrome b; and *rrnS* for small ribosome RNA subunits. tRNA genes are labeled with the single-letter abbreviations of their corresponding amino acids.
