# Supplementary figures and images for: Variation in mitochondrial minichromosome composition between blood-sucking lice of the genus Haematopinus that infest horses and pigs
Source: Parasit Vectors. 2014 Mar 31;7:144. doi: 10.1186/1756-3305-7-144 (PMC4022054; doi:10.1186/1756-3305-7-144)

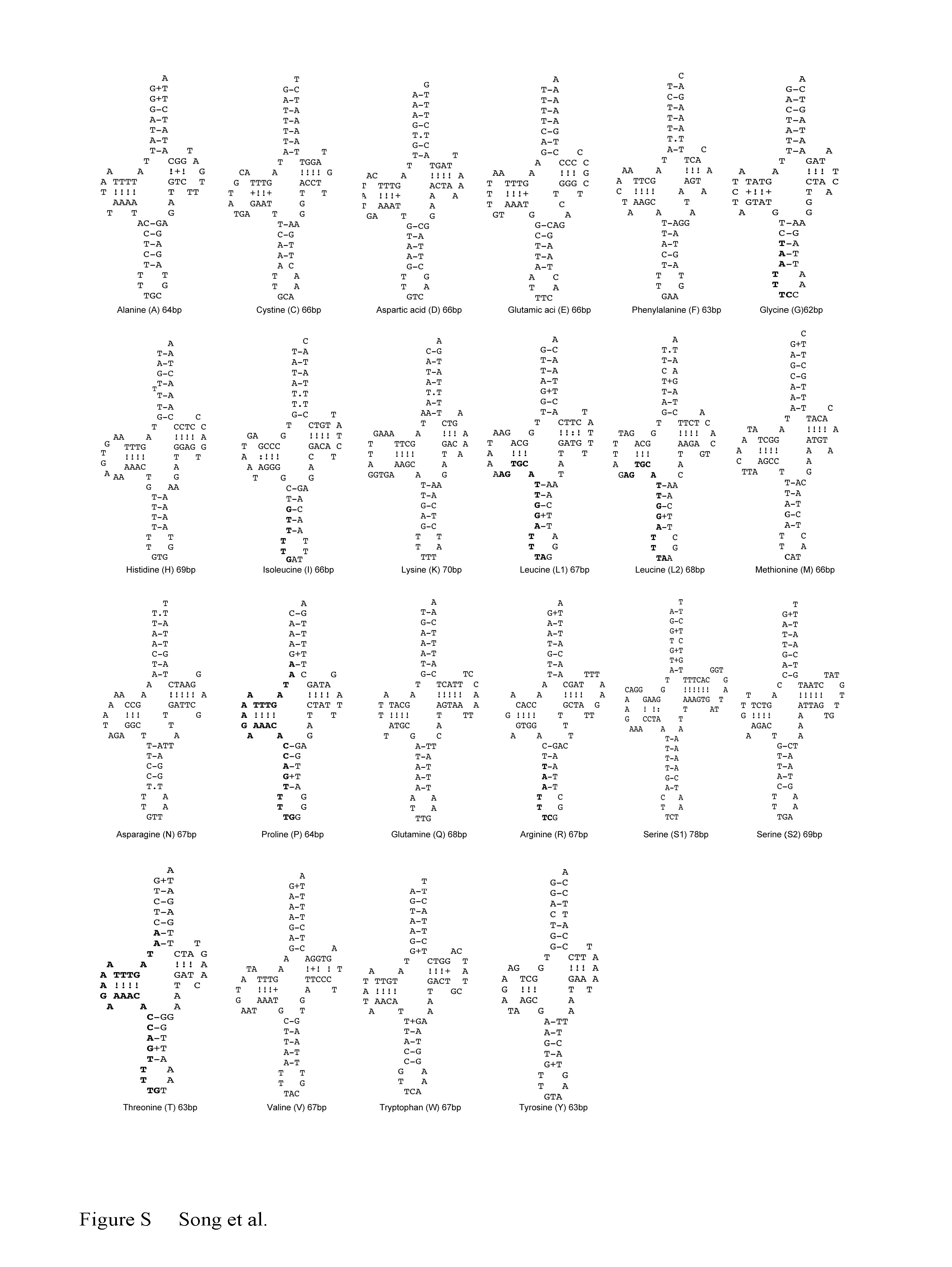

Supplement: Additional file 3 — The inferred secondary structure of the mitochondrial tRNAs of the horse louse, Haematopinus asini. Shared identical sequences between tRNA genes are in bold (see also Table 2). [file 1756-3305-7-144-S3.tiff]
